# Functional Divergence of Microtubule-Associated TPX2 Family Members in *Arabidopsis thaliana*

**DOI:** 10.3390/ijms21062183

**Published:** 2020-03-22

**Authors:** Eva Dvořák Tomaštíková, Twan Rutten, Petr Dvořák, Alisa Tugai, Klara Ptošková, Beáta Petrovská, Daniel van Damme, Andreas Houben, Jaroslav Doležel, Dmitri Demidov

**Affiliations:** 1 Institute of Experimental Botany of the Czech Academy of Sciences, Centre of the Region Hana for Biotechnological and Agricultural Research, Šlechtitelů 31, CZ-77900 Olomouc, Czech Republic; klara.ptoskova@upol.cz (K.P.); petrovska@ueb.cas.cz (B.P.); dolezel@ueb.cas.cz (J.D.); 2 Leibniz Institute of Plant Genetics and Crop Plant Research (IPK), Gatersleben, Corrensstrasse 3, 06466 Seeland, Germany; rutten@ipk-gatersleben.de (T.R.); alisatugai1992@gmail.com (A.T.); houben@ipk-gatersleben.de (A.H.); 3 Department of Botany, Faculty of Science, Palacký University Olomouc, Šlechtitelů 27, CZ-78371 Olomouc, Czech Republic; p.dvorak@upol.cz; 4 Department of Plant Biotechnology and Bioinformatics, Ghent University, 9052 Ghent, Belgium; Daniel.VanDamme@psb.vib-ugent.be; 5 Center for Plant Systems Biology, VIB, 9052 Ghent, Belgium

**Keywords:** *Arabidopsis thaliana*, targeting protein for Xklp2, aurora kinase 1, phylogeny, kinase assay, in vivo co-localization

## Abstract

TPX2 (Targeting Protein for Xklp2) is an evolutionary conserved microtubule-associated protein important for microtubule nucleation and mitotic spindle assembly. The protein was described as an activator of the mitotic kinase Aurora A in humans and the *Arabidopsis* AURORA1 (AUR1) kinase. In contrast to animal genomes that encode only one TPX2 gene, higher plant genomes encode a family with several *TPX2-LIKE* gene members (TPXL). TPXL genes of *Arabidopsis* can be divided into two groups. Group A proteins (TPXL2, 3, 4, and 8) contain Aurora binding and TPX2_importin domains, while group B proteins (TPXL1, 5, 6, and 7) harbor an Xklp2 domain. Canonical TPX2 contains all the above-mentioned domains. We confirmed using in vitro kinase assays that the group A proteins contain a functional Aurora kinase binding domain. Transient expression of *Arabidopsis* TPX2-like proteins in *Nicotiana benthamiana* revealed preferential localization to microtubules and nuclei. Co-expression of AUR1 together with TPX2-like proteins changed the localization of AUR1, indicating that these proteins serve as targeting factors for Aurora kinases. Taken together, we visualize the various localizations of the TPX2-LIKE family in *Arabidopsis* as a proxy to their functional divergence and provide evidence of their role in the targeted regulation of AUR1 kinase activity.

## 1. Introduction

Cell cycle progression and timing of events are crucial for cell division and viability. To ensure equal chromosome segregation during mitosis, the interphase microtubule network represented by the cortical microtubules undergoes a dramatic reorganization and reassembles into a functional mitotic spindle. The spatial and temporal coordination of events is therefore critical. In most cases, such regulation is carried out by post-translational control, such as phosphorylation and dephosphorylation of regulatory proteins. Aurora kinase family of serine/threonine protein kinases are known as very important mitotic kinases evolutionary conserved among eukaryotes [1,2].

The Aurora kinase family of *Arabidopsis thaliana* contains three members that are subdivided into two groups: α (*AURORA1* and *AURORA2*) and β (*AURORA3*). α-Aurora kinases are localized at spindle microtubules during mitosis and cell division plate in cytokinesis, while β-Aurora is localized at centromeres during metaphase [3,4,5]. Interestingly, in human cells, the substitution of just one amino acid, close to the catalytic domain of Aurora A kinase, can functionally convert Aurora A into a B-like Aurora kinase [6,7].

Downregulation of Aurora kinases in *Arabidopsis* results in mitotic cell division defects and interferes with the meristem development [8]. *aurora1/aurora2* double mutant plants display severe defects in formative cell divisions during lateral root formation [5]. Furthermore, *aurora1/aurora3* double mutant plants suffer from aberrant meiosis with the formation of micronuclei, unequal separation of chromosomes and defects in tetrad formation. These defects indicate the importance of Aurora kinases in meiotic cytoskeleton dynamics [9]. Plant α-Auroras have also been shown to affect the microtubule-binding properties of Microtubule-Associated Protein 65-1 (MAP65-1). Phosphorylation control residues in the C-terminal part of MAP65-1 was shown to be important for the efficient cell cycle progression [10,11].

The Targeting Protein for Xklp2 (TPX2), is required to prevent inactivation/dephosphorylation of human Aurora A by phosphatase PP1 at the activation segment T288 [6]. TPX2 was first described as a microtubule-associated protein involved in chromosome-dependent spindle assembly of human cells [12]. During mitosis, TPX2 is released from importin α/β heterodimers due to the high concentration of Ran-GTP at the vicinity of chromosomes. Free TPX2 subsequently activates Aurora kinase A, thus stimulating mitotic spindle assembly in human cells [13]. Homologs of *TPX2* were found in different species, including plants. Similar to the human TPX2, *Arabidopsis* canonical TPX2 contains three conserved domains—the Aurora binding domain responsible for binding and activation of Aurora kinase, the TPX2_importin domain involved in the transfer of protein to the cell nuclei, and the TPX2_Xklp2 motif for binding to microtubules. Further, an additional microtubule-binding motif is present at the N-terminal part of plant TPX2 protein [14]. *Arabidopsis* TPX2 was described as an activator of Aurora1 in vitro [15]. In planta, Aurora1 colocalizes with TPX2 at spindle microtubules throughout mitosis and co-precipitates with TPX2 on microtubules in a cell cycle-specific manner [16].

According to Evrard et al., the N-terminal Aurora binding domain, TPX2_importin, and C-terminal microtubule-binding domain and TPX2_Xklp2 are the conserved domains that characterize true plant *TPX2* orthologues. The authors proposed that other proteins containing only some of the functional blocks may be considered as TPX2-related proteins [17]. In contrast to analyzed genomes of animals, where only one *TPX2* gene was found, *Arabidopsis* encodes 20 genes with sequence homology to some of the domains of canonical TPX2. The first members of the *TPX2-LIKE PROTEIN* (*TPXL*) gene family in *Arabidopsis*, *TPXL2* and *TPXL3*, were functionally characterized only recently [18]. In plants, outside *Arabidopsis*, a *TPXL* gene family containing 12 members was described in *Eucalyptus grandis* [19]. However, the authors focused only on the presence of the TPX2_Xklp2 domain and did not take into consideration proteins with a TPX2_importin domain which is equally important for canonical TPX2 functions.

Here, we revise the phylogeny of the *TPXL* gene family. Based on in silico analyses we identified a group of TPXL proteins with a predicted Aurora kinase binding domain. In vitro kinase assays demonstrated that the Aurora binding domains of *Arabidopsis* TPXL homologs can activate recombinant AUR1. The TPXL members are characterized by different expression patterns and localization, suggesting diversification of *TPXL* genes for specific functions during plant development.

## 2. Results

### 2.1. The A. thaliana Genome Possesses 20 Genes with Similarity to TPX2

**Figure 1:**
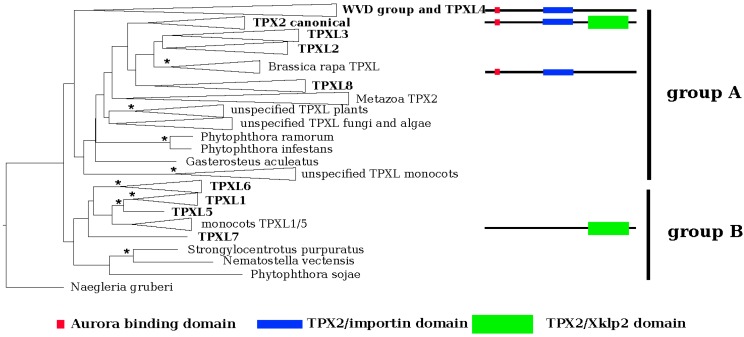
Phylogenetic reconstruction of the TPXL gene family in Arabidopsis. EggNOG4.5 was used to identify orthologs of Arabidopsis canonical Targeting Protein for Xklp2 (TPX2 (At1g03780)). Multiple sequences were performed in MUSCLE 3.8.31 and used for phylogenetic reconstruction of TPX like gene family and the maximum likelihood phylogenetic tree was inferred in RAxML HPC 8.2.9 using the PROTGAMMALG model. The tree topology was tested using ultra-fast bootstrapping by 2000 replicates in IQ-TREE 1.6.9. *Negleria gruberi* TPX2 was selected as an outgroup. Monophyletic clades were collapsed. Consistent with its domain composition, TPXL genes form two separate clusters, groups A and B—proteins containing Aurora binding domain (red square) and importin domain (blue bar) and TPX2_Xklp2 domain (green bar). Significant bootstrap support is represented by an asterisk (* = 99%–100%).

The EggNOG 4.5 database [20] was searched to discover proteins that contain typical TPX2- domains in *Arabidopsis*. In total, 20 proteins were identified, which can be classified into two groups based on their domain composition (Figure 1, Table S1, Figure S1). Group A comprises members with the TPX2_importin domain, while group B members contain the TPX2_Xklp2 domain.

We performed a maximum likelihood phylogenetic analysis with the identified proteins. In agreement with the domain composition, the phylogeny (Figure 1; Figures S1 and S2) revealed two major groups, A and B. Group A could be further divided into two clusters based on the absence (cluster I) or presence (cluster II) of a plant-specific KLEEK-motif. The KLEEK-motif is present in an already characterized group of Wave-Dampened (WVD) and Wave-Dampened-Like (WDL) proteins [21,22] and is typical for microtubule-binding proteins [23] (Figure S2).

The deepest clades (Figure S2) comprised *Arabidopsis* orthologues of TPXL1, TPXL5, TPXL6 and TPXL7 (clades 1, 2, and 3 in [19]). It should be noted that TPXL7 formed a separate clade with only protein sequence from *A. thaliana*. Subsequently derived clade was composed of canonical TPX2 and TPXL2, TPXL3 and TPXL8. The canonical TPX2 clade (clade 3 after [19]) was subdivided into 5 groups representing metazoa, angiosperms, mosses, algae, and fungi. Analyzed representatives of metazoa, algae, and fungi contain a single copy of the canonical TPX2 (Figure S2).

The group of plant-specific proteins with a KLEEK domain (WDLs) contains three major lineages. The first lineage consists of WDLs 5, 7, 9, and TPXL proteins. The second lineage contains WDLs 1, 2, and 3. Finally, the third lineage embraced WDLs 4, 5, and 6. Special attention should be paid to the moss *Physcomitrella patens*, which evolved a unique WDL paralog group (Figure S2). Interestingly, the Aurora binding domain-containing TPXL4 was grouped with the WDL lineage (Figure S2).

Each clade of plant TPX2 and TPXL proteins were divided into two subclades containing monocots and eudicots (Figure S2), thus, there seems to be not a specific paralog for any of these groups.

### 2.2. Functional Prediction of Arabidopsis Aurora Binding Domain

Activation of Aurora A kinases is a significant function of TPX2 proteins. Binding of TPX2 to human Aurora A activates the phosphorylation activity of the kinase and protects it from dephosphorylation by protein phosphatase 2A [6]. Similarly, Aurora kinase binding domain of canonical *Arabidopsis* TPX2 can activate Aurora1 in vitro [15]. Recently we showed that Aurora kinase binding domains of TPXL2 and TPLX3 also can activate Aurora1 in vitro [18]. To characterize the Aurora binding domain of plant group A TPXL proteins, a multiple sequence alignment was performed with orthologues of *Arabidopsis* TPX2. Additionally to TPX2, the *Arabidopsis* genome contains four genes, *TPXL2*, *TPXL3*, *TPXL4*, and *TPXL8* with both TPX2_importin and putative Aurora kinase binding domains (Figure 1).

**Figure 2:**
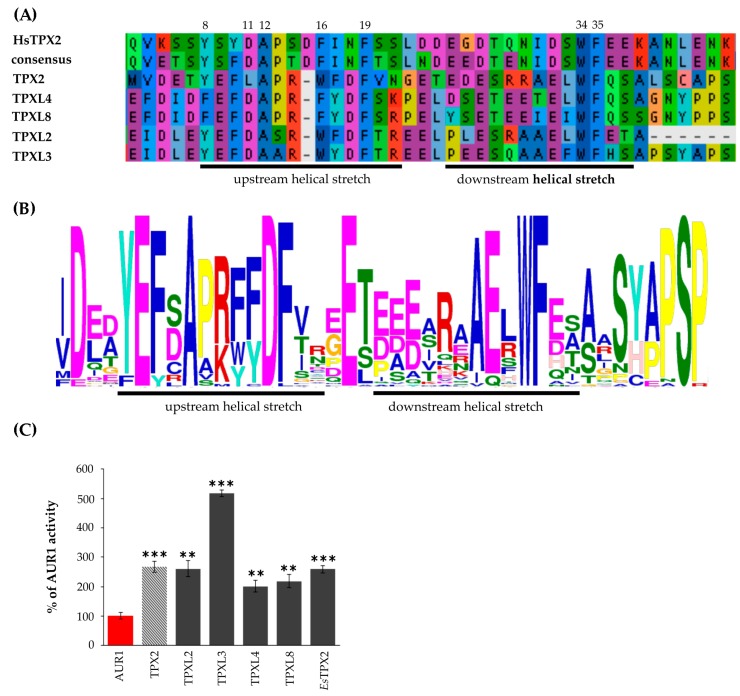
Group A TPXL proteins contain a functional Aurora kinase binding domain. (**A**)—Multiple sequence alignment of putative Aurora binding domains of TPX2, TPXL2, 3, 4 and 8 proteins. TPXL proteins with Aurora binding domain retained all key amino acid residues important for Aurora kinase binding and activation in human (Hs) TPX2. (**B**)—MEME (Multiple Em for Motif Elicitation; [24]) analyses confirmed the presence of similar Aurora binding domain with key conserved residues in 45 proteins from different plants species (**C**)—In vitro kinase assay with recombinant Aurora1 and TPX2 proteins confirmed that all members of TPX2 family with Aurora binding domain can activate Aurora1. TPX2 of distantly related *Eutrema salsugineum* (EsTPX2) also activates *Arabidopsis* Aurora1. *** *p*-value < 0.001 in hypergeometric test, ** *p*-value < 0.01 in hypergeometric test.

The overall sequence conservation is very poor between human TPX2 and plant TPX2-like proteins, but the key residues important for binding of Aurora kinases are evolutionarily conserved (Figure 2A,B). The Aurora binding domain of human TPX2 contains upstream and downstream helical stretches [6]. Despite some amino acid substitutions in the upstream helical stretch of TPXL4/TPXL8 or TPXL2/TPXL3 compared to human TPX2, the hydrophobic side chain involved in stacking interactions is preserved (Figure 2A, amino acid 8). Similarly, the amino acid residues important for Aurora kinase activation (Figure 2A, amino acids 34, 35) [6] are conserved, further indicating the functional conservation of the TPX2/Aurora kinase complex. The upstream helical part of Aurora binding domain of Arabidopsis TPX proteins is enriched with the conservative acidic amino acids E and D compared to Aurora binding domain human TPX2. At the same time, the downstream helical stretch part of Aurora binding domain of plant TPX proteins is characterized by a lower number of acidic AA (Figure 2B). Importantly, MEME (Multiple Em for Motif Elicitation; [24]) analyses confirmed the presence of a similar Aurora binding domain with key conserved residues in 45 proteins from different plant species (Figure 2B, Table S2).

### 2.3. All Arabidopsis TPX2 Family Members Possessing An Aurora Kinase Binding Domain Activate AUR1 In Vitro

The Aurora kinase binding domains of canonical *Arabidopsis* TPX2, TPXL2, and TPXL3 were shown to activate Aurora1 in vitro and increase its phosphorylation activity towards histone H3 as a physiological substrate [15,18]. To address the functionality of the Aurora kinase binding domains of *Arabidopsis* TPXL proteins, we performed in vitro kinase assays. The Aurora binding domains of TPXL2, TPXL3, TPXL4, and TPXL8 were expressed in *Escherichia coli (E. coli)*, purified (Figure S3A) and combined with recombinant AUR1 as enzyme and histone H3 as a substrate. An increase in histone H3 phosphorylation detected by incorporation of radioactive isotope P^32^ into histone H3 was used as a means to measure the activity of AUR1 [25]. The in vitro kinase assay showed that Aurora binding domains of all TPXL proteins can activate AUR1 kinase (Figure 2C, Figure S3B). In addition, this confirms the activation of Aurora through a change in the kinase structure, and not because of the pThr protection in the active center, since phosphatases are absent in the kinase assay.

TPXL3 has the highest activation potential with up to 5-fold increase compared to Aurora1 kinase alone, which was significantly higher than AUR1 activation by canonical TPX2 (Figure 2C).

### 2.4. The Eight Closest Homologs of Canonical TPX2 are Differentially Expressed during Arabidopsis Development

**Figure 3:**
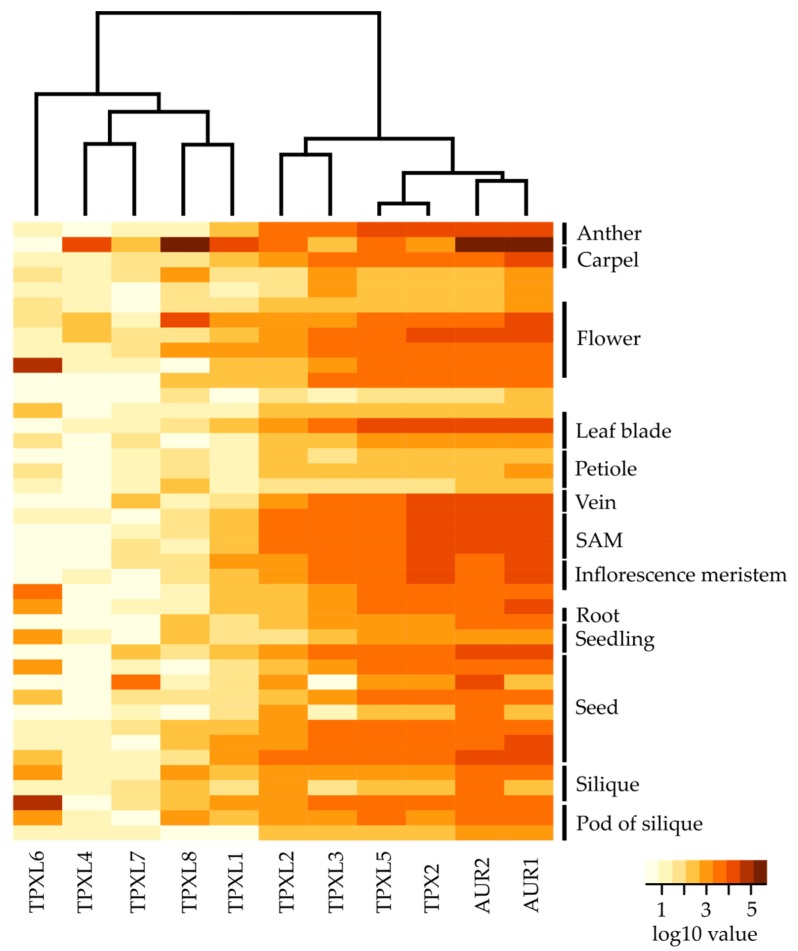
Expression analyses of TPXL and α-Aurora genes at different developmental stages of Arabidopsis. Heat map displays differential expression profiles across various developmental stages. The color bar represents log10 expression values inferred from raw counts of [25]; thereby white color representing the lowest expression values and brown signifies the highest expression level. Black bars indicate a set of multiple samples from the same tissue. The dendrogram was computed and reordered based on gene expression values. Detailed information about tested developmental stages is available in Figure S4 and Table S3.

To investigate the expression of *TPXL* genes, we analyzed publicly available RNA sequencing data from different developmental stages of *Arabidopsis* [26]. To profile gene expression patterns of selected *TPXL* genes, we analyzed the expression across all developmental stages (Figure 3, Figure S4, Table S3). These heat maps illustrate distinct gene expression of *TPXLs* during development. *TPXL2*, *TPXL3*, *TPXL5,* and *TPX2* seem to be the most widely expressed *TPXLs*. In general, expression of *TPX2* was among the highest in all tissues. Strikingly, *TPXL4* was considered as a pseudogene [17]; however, transcriptome analyses showed specific expression of *TPXL4* in mature anthers. *TPXL6* expression was restricted to siliques and *TPXL7* was only detected in dry seeds. Taken together, our data confirmed the validity of our hypothesis that *TPXL* genes might have evolved different functions during plant development. Similar expression patterns for *TPX2*, *TPXL2, TPXL3*, and *TPXL5* point to possible functional redundancy [18]. Moreover, these four proteins are expressed in a similar pattern to *Aurora1* and *Aurora2* in agreement with the fact that they are physiological activators of the kinases.

### 2.5. TPXL Proteins Mostly Localize on Microtubules

**Figure 4:**
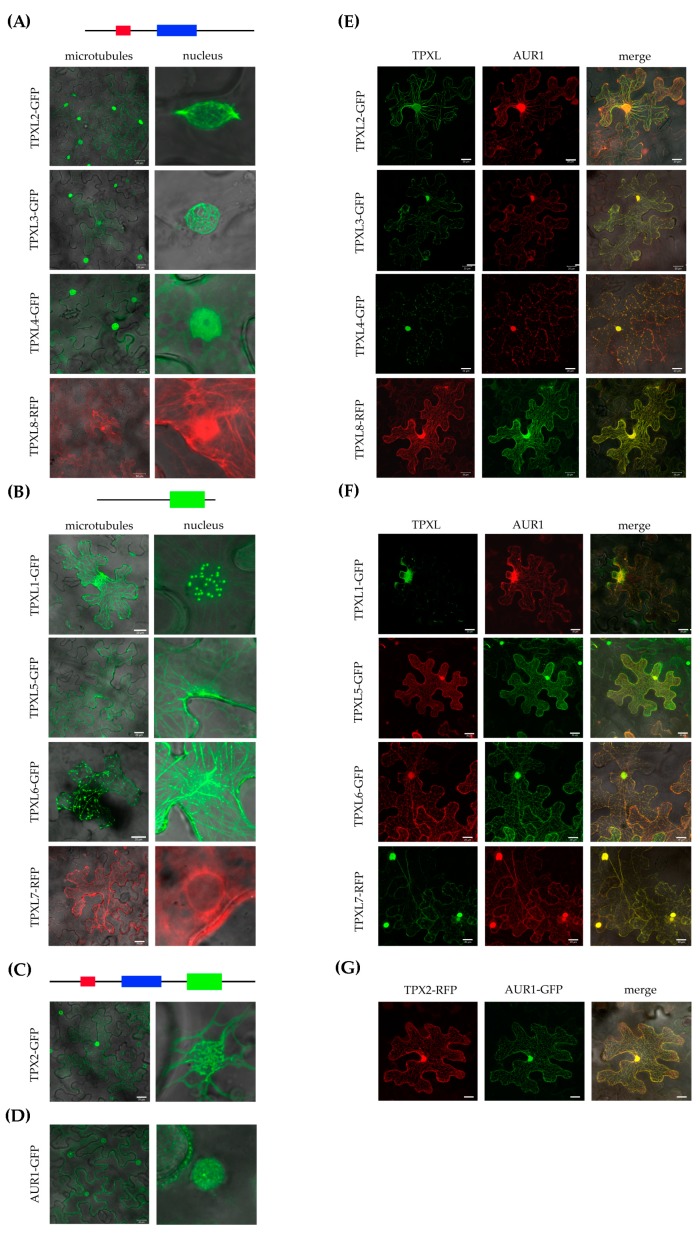
Subcellular localization of Arabidopsis TPXL proteins in tobacco leaf epidermal cells. Images were acquired 2 days after infiltration using a laser scanning confocal microscope. (**A**)—Localization of Group A TPXL proteins on microtubules and in the nucleus. GFP—Green Fluorescent Protein, RFP—Red Fluorescent Protein. (**B**)—Localization of Group B TPXL proteins on microtubules and in the nucleus. (**C**)—Localization of canonical TPX2-GFP. (**D**)—Localization of Aurora1-GFP alone. (**E**)—Co-localization of Group A TPXL and Aurora1. (**F**)—Co-localization of Group B TPXL and Aurora1. (**G**) – Co-localization of TPX2 with Aurora1 as control. Bar = 20 μm.

The canonical TPX2 was shown to localize on microtubular arrays and the TPX2_Xklp2 domain is involved in microtubule binding [14,17]. TPXL proteins of group A (Figure 1) contain a TPX2_importin domain important to interact with alpha importin [27] and an Aurora binding domain. Group B proteins contain a TPX2_Xklp2 domain with a kinesin-targeting signature. To uncover the localization patterns of the selected TPXL proteins, constructs to express fluorescently-labeled translational fusions were infiltrated into *Nicotiana benthamiana (N. benthamiana)* leaves and visualized by confocal microscopy. Consistent with the presence of a TPX2_importin and an Aurora kinase binding domain, group A TPXL proteins showed strong microtubular labeling at the nuclei (Figure 4A). Moreover, the proteins also faintly labeled cortical microtubules (Figure 4A). It should be noted that two members group A TPXL2 and TPXL3 labeled microtubular fibers decorating the nuclear envelope like canonical TPX2 (Figure 4A). Group B TPXL proteins mainly localized with cortical microtubules, TPXL1, TPXL5, and TPXL6 showed very strong labeling resembling cytoskeletal filaments (Figure 4B). TPXL5 also labeled microtubules close to the nucleus (Figure 4B). Interestingly, TPXL7 showed a different localization pattern compared to all other TPXL proteins. TPXL7 lacks microtubular localization and mainly localized in the vicinity of the nuclear membrane (Figure 4B). The canonical TPX2 decorates cytoskeletal cables and bundles of microtubules around nuclei (Figure 4C). These results indicate that TPXL gene family has probably evolved differential targeting and different functions in the regulation of microtubule cytoskeletal dynamics.

### 2.6. TPXL Proteins Re-localize Aurora1 Kinase by Loading It on Microtubular Arrays

During interphase, AUR1 kinase is localized in very low amounts in the nucleus, while the microtubular localization of AUR1 is a hallmark of cell division [5,8]. To check whether TPXL proteins co-localize with AUR1, DNA constructs of fluorescently-labeled fusion variants of AUR1 and TPXL were coinfiltrated into *N. benthamiana* leaves. Normally, AUR1 shows diffuse nuclear and weak cytoplasmic labeling in infiltrated *N. benthamiana* epidermal cells (Figure 4D). Interestingly, after co-infiltration with TPX2 construct, AUR1-GFP is mostly localized on cortical microtubules (Figure 4G). Co-expression of TPXL proteins also re-localized AUR1. TPX2, TPXL1, TPXL2, TPXL3, TPXL4, TPXL6, and TPXL8 re-localize AUR to the nucleus as well as to cortical microtubules, while TPXL4 and TPXL5 relocalize it to microtubules (Figure 4E–G). Interestingly, coexpression of TPXL proteins with AUR1 not only changed the localization of the kinase but also re-localized some TPXL proteins. The most striking redistribution was observed for TPXL7, which shared a strong nuclear localization with AUR1. These results indicate that colocalization of TPXL proteins with AUR1 is not only dependent on the presence of a functional Aurora binding domain, but other mechanisms must exist to regulate these proteins.

## 3. Discussion

### 3.1. Diversity of TPX in Plants

TPX2 is a widely conserved microtubule-associated protein required for mitotic spindle assembly and function [12,14] although recent findings also show its involvement in DNA damage response [28,29]. Functions of TPX2 are well characterized in animals including humans; however, the knowledge from plant systems is sparse. Moreover, unlike animals that contain a single TPX2 gene, plants contain a family of twenty TPX2-related proteins. In this work, we performed phylogenetic analyses of the whole group of *Arabidopsis* TPXL proteins. The canonical TPX2 contains an Aurora binding domain, a TPX2_importin, and TPX2_Xklp2 domains. Only the presence of these three domains defines the bona fide homologs of TPX2 [17]. In plants, TPXL proteins have been also identified in *Eucalyptus grandii* [19]; however, the authors did not take into account the importance of the TPX2_importin domain and therefore missed the entire group A. Consequently, we show the presence of 16 proteins with a TPX2_Xklp2 domain and 4 proteins with TPX2_importin and Aurora binding domains in the *A. thaliana* genome. Strikingly, all proteins containing the TPX2_importin domain also contain the Aurora binding domain.

In agreement with the predicted domain composition, group A TPXL proteins combine an Aurora binding domain and also a TPX2_importin domain. The functional relevance of the lack of the TPX2_Xklp2 domain compared to TPX2_importin remains to be determined. Group B proteins with TPX2_Xklp2 were all clustered together in a separate clade. Despite the presence of an Aurora binding domain, TPXL4 clustered together with the plant-specific lineage of WVD proteins containing the KLEEK domain and seems to be an ancestor of the WDL clade. Further sequence analyses did not reveal the presence of a KLEEK motif in TPXL4. This may cause a slight difference in TPXL4 function and therefore explain different localization of TPXL4 compared to other members of group A TPXLs. Possible explanations for the diversification of the TPXL gene family in plants could be differences in the organization of microtubule-organizing centers in animals and plants. Some of the diversity also comes from whole-genome duplications as suggested by the similar exon-intron structure of some of the TPXL genes (Figure S5). It should also be noted that compared to animals, plants usually encode two genes of α-Aurora kinases [5]. Although we do not have any data about the specificity of complex formation between different α-Auroras and TPXL proteins during development we have already shown the interaction of AUR1 and AUR2 with TPXL2 and TPXL3 [18]. On the other hand, unlike animals, plants show a high level of endopolyploidization, which is highly specific for different tissues and different stages of development. As the activation of α-Aurora kinases and regulation of spindle assembly play a key role in endopolyploidization this can explain the high number of TPXL genes in plant genome. Furthermore, the complex TPX2 gene family could represent a land plant adaptation strategy of the spindle assembly and positioning [30].

In addition to the canonical TPX2, we have described four additional Aurora binding domain-containing proteins. Until now, the canonical TPX2 of *Arabidopsis* was considered to consist of two adjacent Aurora kinase binding domains [14]. However, crystallographic analyses of the N-terminal part of human TPX2 with the Aurora A catalytic domain showed the presence of two helical stretches separated with a short linker that is responsible for the interaction [6]. Indeed, we were able to identify those two stretches in the Aurora binding domain of plant TPXLs. Although the overall plant sequences of the Aurora binding domain are highly divergent from the consensus sequence, residues important for binding, and activation of the Aurora kinase in plant TPXL are highly conserved. Interestingly, the Aurora kinase binding domains of plants TPX2/TPXL, like in the case of animal TPX2 also localize at the N-terminus. Whether this is because of the sterical reasons or functional diversification remains to be determined.

Based on the domain analysis of TPXL proteins, we observe a possible specialization of TPXL. Group A, without the kinesin domain [18] might be implicated in the regulation of AUR1 and group B in the regulation of kinesins in *Arabidopsis.* It seems that during the evolution plants divided TPXL members depending on the tissue specificity and developmental stage. Importantly, Aurora binding domain is always present together with the TPX2_importin binding domain. This allows importin to bind TPXs of group A and prevent activation of α-Aurora kinases [14,27]. This raises an additional level of regulation of α-Aurora kinases. The presence of a larger number of kinesin genes in plant genomes, in comparison to animals or humans [31,32] can explain the diversity of TPXL isoforms in the B group. At the same time, the reason for such evolutionary separation of the TPXL family based on functionality remains unclear.

### 3.2. Activation of Aurora Kinase by TPX2 Family Proteins Seems to Be Evolutionary Conserved

Aurora kinases are known to phosphorylate various targets. However, in plants only histone H3 [25], MAP65-1 [10], microtubule-associated proteins TPX2 [15], TPXL2, and TPXL3 [18] were confirmed as targets of Aurora1 kinase. Additionally, several transcription factors were shown to be phosphorylated by both AUR1 and AUR3 kinases [33]. Several of these transcription factors are closely related to the regulation of developmental processes. It is tempting to speculate that phenotypical similarities of both AUR1 and TPX2 mutants [5,8,16] are partially dependent on the activation of the AUR1 kinase by TPX2 family proteins. The in vitro kinase assay proved the activation potential of the Aurora binding domain of TPXL proteins on recombinant AUR1. TPXL3 was the strongest activator of Aurora1 with even higher activity than the canonical TPX2. Importantly, activation of Aurora kinase by TPX2 seems to be evolutionary conserved, as the Aurora binding domain of TPX2 from a distantly related *Brassicaceae* species *Eutrema salsugineum* is also capable of Aurora1 activation (Figure 2C, Figure S3).

### 3.3. TPXL Members with Aurora Binding Domain are Strongly Expressed

Although all the tested proteins were able to activate AUR1, not much was known about their expression during *Arabidopsis* development. Group A TPXL proteins showed generally stronger expression compared to group B. It is therefore possible, that plants need more TPXL proteins with Aurora binding domain. Despite this, there is always one protein that seems to have a generally higher expression and could probably fulfill housekeeping function. Consistently with the previous findings, TPX2 is expressed in highly dividing tissues such as SAM and inflorescence meristem. Cells are actively dividing in SAM and expression of TPXL proteins correlates with their expected role in planta. The high expression of TPX2 is in agreement with the importance of AUR1 to phosphorylate various substrates, such as histone H3 during cell division [3] in *Arabidopsis* and during gametophyte development to phosphorylate CENH3 [34]. Moreover, the expression pattern of AUR1 and AUR2 is highly similar to that of TPX2, TPXL2, and TPXL3, suggesting a common regulation of these proteins. TPXL2 and TPXL3 were shown as interactors of AUR1 and AUR2 and TPXL3 is a primary activator of Aurora1 [18] further supporting the importance of their common expression patterns.

### 3.4. The Localization of TPXL is Associated with Their Importance for Spindle Microtubules

We also speculate that activation of Aurora kinase by TPX2-related proteins TPXL2, TPXL3, TPXL4, and TPXL8 is related to its nuclear localization which is in agreement with the proposed function of TPX2 in chromatin-induced mitotic spindle assembly [16]. On the other hand, we were not sure that TPXL without TPX2_Xklp2 domain can be localized on microtubules. Most likely similarly to canonical TPX2, other TPXL proteins contain a microtubule-binding domain in front of the TPX2_importin domain [17]. Infiltration of *N. benthamiana* confirmed the functionality of the tested TPXL proteins. Most of the proteins localized both in the nucleus and on microtubules. It has previously been shown that the overexpression of TPX2 in *Arabidopsis* results in the nuclear envelope and nuclear localization [16]. Relatively similar localization was also observed for other members of the TPXL gene family.

Co-localization analyses showed especially strong overlap with AUR1 kinase for those TPXL with an Aurora binding domain. In humans, the interaction between TPX2 and Aurora A is not only important for its activation but also for targeting of the kinase to the spindle microtubules [35] and assembly of the spindle of the correct length [36]. Apparently, in plant cells, along with the relocalization of inactive Aurora kinase from the cytoplasm to microtubules using TPX proteins, simultaneous local activation of Aurora kinase occurs. The presence of active Aurora on microtubules could be related to its active function in *Arabidopsis*: Spindle Assembly Checkpoint (SAC) regulation, spindle organization/orientation, regulation of microtubule polymerization or depolymerization and formation of centrosome-like structures.

Importantly, most of the TPXL proteins changed the localization of AUR1. This was a big surprise for us because it is unclear how TPXL isoforms without Aurora binding domain can interact with Aurora kinases. This phenomenon could be explained by the presence of the coiled-coil motif, a domain involved in protein dimerization and protein–protein interactions. The coiled-coil motif is present in all TPXL proteins. Importantly, human TPX2 is known to provide a scaffold for the chromosome passenger complex [37]. Similarly, TPXL might have a critical role in the recruitment of the microtubule nucleation complex by Aurora kinase.

## 4. Material and Methods

### 4.1. Identification of TPXL Proteins

The BLAST (Basic Local Alignment Search Tool, [38]) was used to identify protein homologs of canonical TPX2 protein (At1g03780) in *Arabidopsis*. To characterize the domain composition of TPXL proteins, in silico analyses of protein sequences using PFAM30 [39] and SMART (Simple Modular Architecture Research Tool, [40]) domain prediction programs were performed.

### 4.2. Phylogenetic Analysis

The EggNOG4.5 database (http://eggnogdb.embl.de/; [20]) was used to identify orthologs of TPX2_Xklp2 and TPX2_importin domain-containing proteins. The database EggNOG4.5 contains an orthologous group of genes, which were retrieved from eukaryotic, prokaryotic and viral sequencing projects. Each identified homolog of TPX2 (At1g03780) in *Arabidopsis* was submitted to search separately. Some identical sequences occurred in several orthologous groups and those were removed. The final dataset contained 458 protein sequences. Subsequently, multiple sequence alignment was performed in MUSCLE 3.8.31 [41]. The maximum likelihood phylogenetic tree was inferred in RAxML HPC 8.2.9 (https://cme.h-its.org/exelixis/web/software/raxml/; [42]) using PROTGAMMALG model via Cipres Science Gateway [43]. The tree topology was tested using ultra-fast bootstrapping by 2000 replicates in IQ-TREE 1.6.9 [44,45]. The tree was rooted in the TPX2 protein of *Naegleria gruberi. Naegleria gruberi* belongs to excavates. This group of protists is classified outside of all other organisms in the tree. Thus, it serves as an ideal outgroup taxon.

The maximum likelihood phylogenetic analysis of TPX2-like proteins in *A. thaliana* was performed in the MEGA 7 (GAMMA+LG model; [46]). The tree topology was tested using bootstrapping by 1000 replicates.

### 4.3. Gene differential Expression Analyses

Med normalized raw counts of *A. thaliana* gene expression data were downloaded from [26]. Obtained raw counts were further within-sample normalized using a transcript per million (TPM) [47]. Data were log-normalized and used for hierarchical clustering using function heatmap.2 in R [48] (Table S3).

### 4.4. Plant Material

*A. thaliana* ecotype Columbia plants were used in this study, originally obtained from the European *Arabidopsis* Stock Centre (NASC ID: N3176). Plants were grown in growth chambers under short day conditions and after 2 weeks cultivated under long day conditions at 20 °C. *N. benthamiana* plants (Accession number: NIC 660, IPK Gatersleben, Germany) were grown under a 12 h photoperiod at a constant temperature of 26 °C.

### 4.5. Cloning of TPXL Genes

TPXL sequences were obtained by PCR amplification from *Arabidopsis* cDNA or gDNA using Platinum Pfx DNA Polymerase (Thermo Fisher Scientific, Prague, Czech Republic) using primers listed in Table S4. The amplified fragments were cloned into a Gateway donor vector pDONR207 as described previously [49]. cDNA or gDNA of TPXL genes were subsequently cloned as a fusion with Green or Red Fluorescent Proteins (GFP and RFP) into a Gateway destination vectors pH7FWG2.0 and pH7WGF2.0 for N- and C- terminal GFP fusion and pH7RWG2.0 and pH7WGR2.0 for RFP fusions. For expression of the recombinant Aurora binding domain, the first 300 bp of respective TPXL genes, comprising the Aurora binding domain, were cloned as 6xHis fusion into a pET55DEST expression vector using primers listed in Table S4.

### 4.6. Production of Recombinant Proteins

GST-Aurora1 was expressed in *E. coli* C-43 strain (Lucigen, www.lucigen.com) and purified under native conditions as described in [15]. Aurora binding domains of TPXL genes were expressed in *E.coli* BL-21 (GE Healthcare Life Sciences, https://www.gelifesciences.com) and purified under denaturing conditions as described in [25].

### 4.7. In Vitro Kinase Assay

Purified recombinant proteins were desalted in kinase buffer using 7K MWCO Zeba Spin Columns (Thermo Scientific) and processed as described in [15]. Briefly, samples with Aurora1 were incubated at 30 °C, 30 min with 0.1 mM ATP for activation of the kinases. Subsequently, [^32^P]ATP and substrates (*Arabidopsis* histone H3 ~10 µg, common substrate of Aurora1; [25]) were added and incubated for an additional 60 min at 30 °C.

### 4.8. Infiltration of N. benthamiana and Confocal Microscopy

Transient infiltration of *N. benthamiana* leaf cells was performed as described in [50]. For the infiltration of multiple constructs, bacterial cultures with an OD between 1–1.3 were mixed in a 1:1 ratio. Expression of GFP and RFP was analyzed by a Zeiss LSM780 confocal laser scanning microscope (Carl Zeiss, Jena, Germany) using a 20× NA 0.8 objective for overview recordings and a 40× NA 1.2 water-emersion objective for detailed recordings of nuclei. Before recording, expression of GFP and RFP was confirmed by photospectrometric analysis. Emission of GFP was recorded using a 488 nm laser line in combination with a 490–540 nm bandpass emission, RFP was excited with a 561 nm laser line and emission measured using a 570–620 nm bandpass. The distribution of fluorescence signals within the nucleus was recorded as Z-stacks. For a colocalization analysis, probes were excited with dual 488 nm and 561 nm laser lines in combination with a 488/561 nm beam splitter.

### 4.9. Accession Numbers

Sequence data from this article can be found in the EMBL/GenBank data library under following accession numbers Q5XVC4 (AGI locus identifier At3g01015, TPXL1), Q4V3B0 (At4g11990, TPXL2), Q4V3C5 (At4g22860, TPXL3), F4K6K7 (At5g07170, TPXL4), F4K9U0 (At5g15510, TPXL5), F4K773 (At5g37478, TPXL6), Q9FKW1 (At5g44270, TPXL7), Q5XUX8 (At5g62240, TPXL8), and F4I2H7 (At1g03780, TPX2) (Table S1).

## Supplementary Materials

Supplementary Materials can be found at https://www.mdpi.com/1422-0067/21/6/2183/s1. Figure S1. EggNOG4.5 was used to identify orthologs of *Arabidopsis* canonical TPX2 (At1g03780). Multiple sequence alignment was performed in MUSCLE 3.8.31. Available online: https://figshare.com/articles/Untitled_Item/8798357. Figure S2. Phylogenetic reconstruction of the TPXL gene family. EggNOG4.5 was used to identify orthologs of *Arabidopsis* canonical TPX2 (At1g03780). Multiple sequence alignment was performed in MUSCLE 3.8.31 and used for phylogenetic reconstruction of TPX like gene family and the maximum likelihood phylogenetic tree was inferred in RAxML HPC 8.2.9 using PROTGAMMALG model. The tree topology was tested using rapid bootstrapping by 500 replicates. *Negleria gruberi* TPX2 was used as an outgroup. Consistently with its domain composition, TPXL genes form two separate clusters, groups A and B – proteins containing Aurora binding domain (red square) and importin domain (blue bar) and TPX2_Xklp2 domain (green bar). Figure S3. Aurora binding domains of TPX2-like proteins increase the activity of Aurora1. (A) Expression, purification, and quantification of Aurora binding domains of TPX2-like proteins. BSA was used as a standard for quantification. (B) Aurora1 phosphorylation activity towards histone H3 increased after the addition of recombinant Aurora binding domains of different TPX2 like proteins. Radioactive kinase assay was performed with the first 100 amino acids at the N-terminus of TPX proteins. Figure S4. Expression analyses of TPXL and α-Aurora gene family during *Arabidopsis* development. Heat map and hierarchical cluster display differential expression profiles across various developmental stages (extended version of Figure 3). The color bar represents log10 expression values inferred from raw counts of [26]; thereby white color representing the lowest expression values and brown signifies the highest expression level. The dendrogram was computed and reordered based on gene expression values. Figure S5. Similar exon-intron structure of some of the TPXL genes. Examples of gene duplications based on high similarity in the exon-intron structure of investigated genes, according to NCBI Database. Table S1. Gene names, UniProt identifiers and transcript lengths of genes used in this study. Table S2. List of Uniprot identifiers of plant TPXL proteins used for prediction of Aurora kinase binding domain. Table S3. Log10 expression values of TPXL genes and α-Aurora kinases from RNA-seq [26] and a complete list of *Arabidopsis* developmental stages used for generation of the heatmaps. Table S4. List of primers used in this study.

## Abbreviations

ATP	Adenosine TriPhosphate
AUR1	Aurora1
AUR2	Aurora2
BLAST	Basic Local Alignment Search Tool
CENH3	Centromeric Histone 3
*E. coli*	*Escherichia coli*
eGFP	Enhanced Green Fluorescent Protein
GFP	Green Fluorescent Protein
GST	Glutathione S-Transferase
H3	Histone 3
KLEEK	Short motif of amino acids lysine, leucine, glutamic acid, glutamic acid, lysineS
MAP65	Microtubule-Associated Protein 65
*N. benhamiana*	*Nicotiana benthamiana*
OD	Optical Density
PCR	Polymerase Chain Reaction
RFP	Red Fluorescent Protein
TPX2	Targeting Protein for Xklp2
TPXL (1–8)	Targeting Protein for Xklp2-Like (1–8)
WVD	Wave-Dampened
WDL	Wave-Dampened Like

## References

[1] Willems E., Dedobbeleer M., Digregorio M., Lombard A., Lumapat P.N., Rogister B. (2018). The functional diversity of Aurora kinases: A comprehensive review. Cell Div..

[2] Weimer A.K., Demidov D., Lermontova I., Beeckman T., Van Damme D. (2016). Aurora kinases throughout plant development. Trends Plant Sci..

[3] Demidov D. (2005). Identification and dynamics of two classes of aurora-like kinases in arabidopsis and other plants. Plant Cell.

[4] Kawabe A., Matsunaga S., Nakagawa K., Kurihara D., Yoneda A., Hasezawa S., Uchiyama S., Fukui K. (2005). Characterization of plant Aurora kinases during mitosis. Plant Mol. Biol..

[5] Van Damme D., De Rybel B., Gudesblat G., Demidov D., Grunewald W., De Smet I., Houben A., Beeckman T., Russinova E. (2011). Arabidopsis α Aurora kinases function in formative cell division plane orientation. Plant Cell.

[6] Bayliss R., Sardon T., Vernos I., Conti E. (2003). Structural basis of Aurora-A activation by TPX2 at the mitotic spindle. Mol. Cell.

[7] Fu J., Bian M., Liu J., Jiang Q., Zhang C. (2009). A single amino acid change converts Aurora-A into Aurora-B-like kinase in terms of partner specificity and cellular function. Proc. Natl. Acad. Sci. USA.

[8] Petrovská B., Cenklová V., Pochylová Ž., Kourová H., Doskočilová A., Plíhal O., Binarová L., Binarová P. (2012). Plant Aurora kinases play a role in maintenance of primary meristems and control of endoreduplication. New Phytol..

[9] Demidov D., Lermontova I., Weiss O., Fuchs J., Rutten T., Kumke K., Sharbel T.F., Van Damme D., De Storme N., Geelen D. (2014). Altered expression of Aurora kinases in Arabidopsis results in aneu- and polyploidization. Plant J..

[10] Boruc J., Weimer A.K., Stoppin-Mellet V., Mylle E., Kosetsu K., Cedeño C., Jaquinod M., Njo M., De Milde L., Tompa P. (2017). Phosphorylation of MAP65-1 by Arabidopsis Aurora kinases is required for efficient cell cycle progression. Plant Physiol..

[11] Smertenko A.P., Chang H.-Y., Sonobe S., Fenyk S.I., Weingartner M., Bögre L., Hussey P.J. (2006). Control of the AtMAP65-1 interaction with microtubules through the cell cycle. J. Cell Sci..

[12] Gruss O.J., Wittmann M., Yokoyama H., Pepperkok R., Kufer T., Silljé H., Karsenti E., Mattaj I.W., Vernos I. (2002). Chromosome-induced microtubule assembly mediated by TPX2 is required for spindle formation in HeLa cells. Nat. Cell Biol..

[13] Gruss O.J., Vernos I. (2004). The mechanism of spindle assembly: Functions of Ran and its target TPX2. J. Cell Biol..

[14] Vos J.W., Pieuchot L., Evrard J.-L., Janski N., Bergdoll M., de Ronde D., Perez L.H., Sardon T., Vernos I., Schmit A.C. (2008). The plant TPX2 protein regulates prospindle assembly before nuclear envelope breakdown. Plant Cell.

[15] Tomaštíková E., Demidov D., Jeřábková H., Binarová P., Houben A., Doležel J., Petrovská B. (2015). TPX2 protein of Arabidopsis activates Aurora kinase 1, but not Aurora kinase 3 in vitro. Plant Mol. Biol. Report..

[16] Petrovská B., Jeřábková H., Kohoutová L., Cenklová V., Pochylová Ž., Gelová Z., Kočárová G., Váchová L., Kurejová M., Tomaštíková E. (2013). Overexpressed TPX2 causes ectopic formation of microtubular arrays in the nuclei of acentrosomal plant cells. J. Exp. Bot..

[17] Evrard J., Pieuchot L., Vos J.W., Vernos I., Schmit A. (2009). Plant TPX2 and related proteins. Plant Signal Behav..

[18] Boruc J., Deng X.-G., Mylle E., Besbrugge N., Van Durme M., Demidov D., Tomaštíková E.D., Tan T.R.C., Vandorpe M., Eeckhout D. (2019). TPX2-LIKE PROTEIN 3 is the primary activator of α Aurora kinases and is essential for embryogenesis. Plant Physiol..

[19] Du P., Kumar M., Yao Y., Xie Q., Wang J., Zhang B., Gan S., Wang Y., Wu A.M. (2016). Genome-wide analysis of the TPX2 family proteins in Eucalyptus grandis. BMC Genom..

[20] Huerta-Cepas J., Szklarczyk D., Forslund K., Cook H., Heller D., Walter M.C., Rattei T., Mende D.R., Sunagawa S., Kuhn M. (2016). EGGNOG 4.5: A hierarchical orthology framework with improved functional annotations for eukaryotic, prokaryotic and viral sequences. Nucleic Acids Res..

[21] Liu X., Qin T., Ma Q., Sun J., Liu Z., Yuan M., Mao T. (2013). Light-Regulated Hypocotyl Elongation Involves Proteasome-Dependent Degradation of the Microtubule Regulatory Protein WDL3 in Arabidopsis. Plant Cell.

[22] Yuen C.Y.L., Pearlman R.S., Silo-Suh L., Hilson P., Carroll K.L., Masson P.H. (2014). WVD2 and WDL1 modulate helical organ growth and anisotropic cell expansion in Arabidopsis. Plant Physiol..

[23] Perrin R.M., Wang Y., Yuen C.Y.L., Will J., Masson P.H. (2007). WVD2 is a novel microtubule-associated protein in *Arabidopsis thaliana*. Plant J..

[24] Bailey T.L., Boden M., Buske F.A., Frith M., Grant C.E., Clementi L., Ren J., Li W.W., Noble W.S. (2009). MEME Suite: Tools for motif discovery and searching. Nucleic Acids Res..

[25] Demidov D., Hesse S., Tewes A., Rutten T., Fuchs J., Karimi Ashtiyani R., Lein S., Fischer A., Reuter G., Houben A. (2009). Aurora1 phosphorylation activity on histone H3 and its cross-talk with other post-translational histone modifications in Arabidopsis. Plant J..

[26] Klepikova A.V., Kasianov A.S., Gerasimov E.S., Logacheva M.D., Penin A.A. (2016). A high resolution map of the arabidopsis thaliana developmental transcriptome based on RNA-seq profiling. Plant J..

[27] Schatz C., Santarella R., Hoenger A., Karsenti E., Mattaj I.W., Gruss O.J., Carazo--Salas R.E. (2003). Importin alpha-regulated nucleation of microtubules by TPX2. EMBO J..

[28] Neumayer G., Nguyen M.D. (2014). TPX2 impacts acetylation of histone H4 at lysine16: Implications for DNA damage response. PLoS ONE..

[29] Neumayer G., Belzil C., Gruss O.J., Nguyen M.D. (2014). TPX2: Of spindle assembly, DNA damage response, and cancer. Cell Mol. Life Sci..

[30] Yamada M., Goshima G. (2017). Mitotic spindle assembly in land plants: Molecules and mechanisms. Biology.

[31] Gicking A.M., Swentowsky K.W., Dawe R.K., Qiu W. (2018). Functional diversification of the kinesin-14 family in land plants. FEBS Lett..

[32] Zhu C., Dixit R. (2012). Functions of the Arabidopsis kinesin superfamily of microtubule-based motor proteins. Protoplasma.

[33] Takagi M., Sakamoto T., Suzuki R., Nemoto K., Obayashi T., Hirakawa T., Matsunaga T.M., Kurihara D., Nariai Y., Urano T. (2016). Plant Aurora kinases interact with and phosphorylate transcription factors. J. Plant Res..

[34] Demidov D., Heckmann S., Weiss O., Rutten T., Dvořák Tomaštíková E., Kuhlmann M., Patrick Scholl P., Municio C.M., Lermontova I., Houben A. (2019). Deregulated Phosphorylation of CENH3 at Ser65 Affects the Development of Floral Meristems in *Arabidopsis thaliana*. Front Plant Sci..

[35] Kufer T.A., Silljé H.H.W., Körner R., Gruss O.J., Meraldi P., Nigg E.A. (2002). Human TPX2 is required for targeting aurora-a kinase to the spindle. J. Cell Biol..

[36] Bird A.W., Hyman A.A. (2008). Building a spindle of the correct length in human cells requires the interaction between TPX2 and Aurora A. J. Cell Biol..

[37] Iyer J., Tsai M.Y. (2012). A novel role for TPX2 as a scaffold and co-activator protein of the Chromosomal Passenger Complex. Cell Signal..

[38] Altschul S.F., Gish W., Miller W., Myers E.W., Lipman D.J. (1990). Basic local alignment search tool. J. Mol. Biol..

[39] Finn R.D., Tate J., Mistry J., Coggill P.C., Sammut S.J., Hotz H.-R. (2008). The Pfam protein families database. Nucleic Acids Res..

[40] Letunic I., Doerks T., Bork P. (2011). SMART 7: Recent updates to the protein domain annotation resource. Nucleic Acids Res..

[41] Edgar R.C. (2004). MUSCLE: Multiple sequence alignment with high accuracy and high throughput. Nucleic Acids Res..

[42] Stamatakis A. (2014). RAxML version 8: A tool for phylogenetic analysis and post-analysis of large phylogenies. Bioinformatics.

[43] Miller M.A. Creating the CIPRES Science gateway. Proceedings of the 2010 Gateway Computing Environments Workshop (GCE).

[44] Hoang D.T., Chernomor O., von Haeseler A., Minh B.Q., Vinh L.S. (2018). UFBoot2: Improving the Ultrafast Bootstrap Approximation. Molecular biology and evolution. Mol. Biol. Evol..

[45] (2015). IQ-TREE: A fast and effective stochastic algorithm for estimating maximum-likelihood phylogenies. Mol. Biol. Evol..

[46] Kumar S., Stecher G., Tamura K. (2016). MEGA7: Molecular Evolutionary Genetics Analysis version 7.0 for bigger datasets. Mol. Biol. Evol..

[47] Conesa A., Madrigal P., Tarazona S., Gomez-Cabrero D., Cervera A., McPherson A., Szcześniak M.W., Gaffney D.J., Elo L.L., Zhang X. (2016). A survey of best practices for RNA-seq data analysis. Genome Biol..

[48] R Core Team (2017). R: A Language and Environment for Statistical Computing.

[49] Tomaštíková E., Cenklová V., Kohoutová L., Petrovská B., Váchová L., Halada P., Kočárová G., Binarová P. (2012). Interactions of an Arabidopsis RanBPM homologue with LisH-CTLH domain proteins revealed high conservation of CTLH complexes in eukaryotes. BMC Plant Biol..

[50] Phan H.T., Conrad U. (2016). Vaccine Technologies for Veterinary Viral Diseases.

