# Supplementary material for: Functional Divergence of Microtubule-Associated TPX2 Family Members in Arabidopsis thaliana
Source: Int J Mol Sci. 2020 Mar 22;21(6):2183. doi: 10.3390/ijms21062183 (PMC7139753; doi:10.3390/ijms21062183)
Supplement: Supplementary file 1 [file ijms-21-02183-s001.zip › Figure S3.pdf]

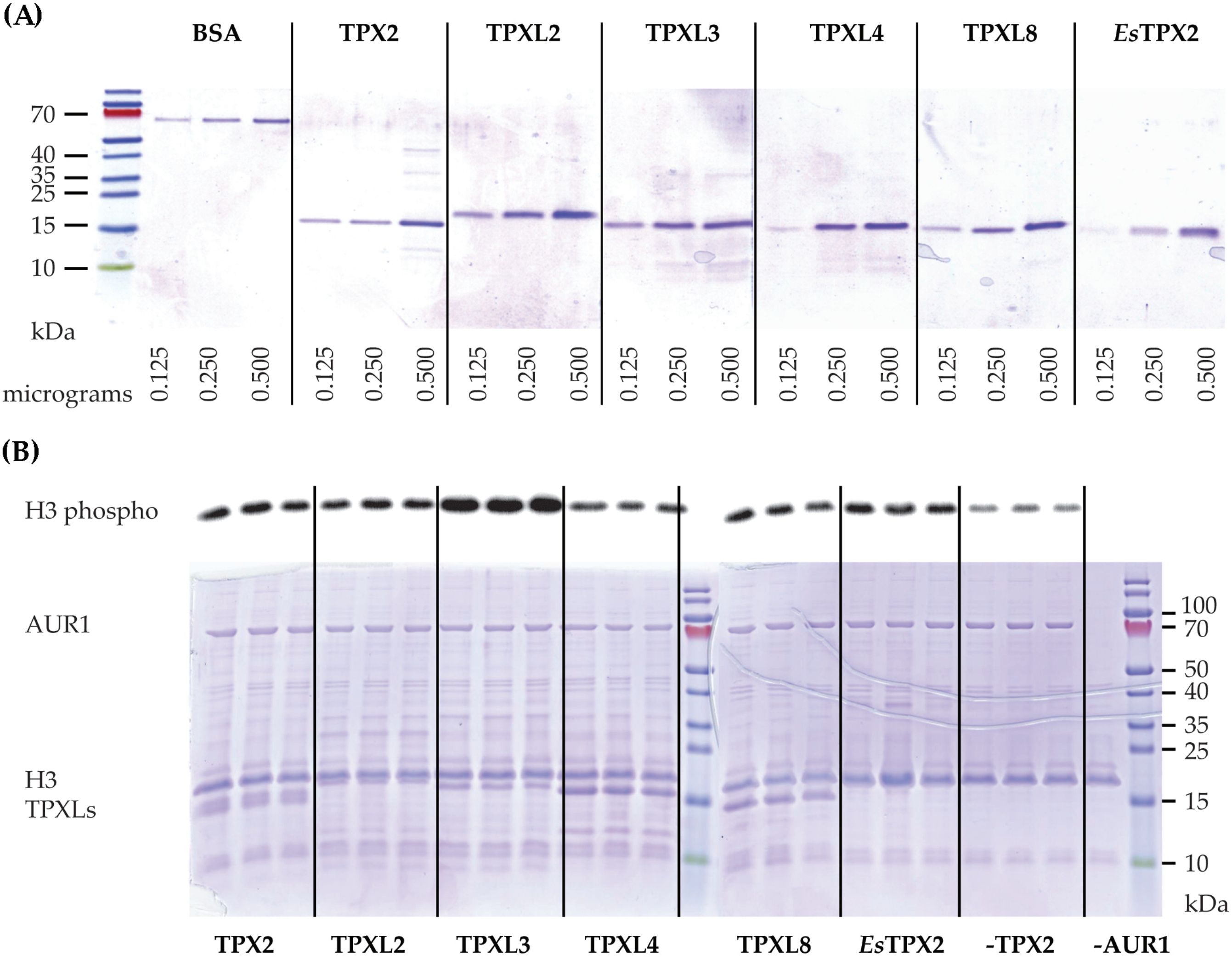

Figure S3: Aurora binding domains of TPX2-like proteins increase the activity of Aurora1. A – Expression, purification and quantification of Aurora binding domains of TPX2-like proteins. BSA was used as a standard for quantification. B - Aurora1 phosphorylation activity towards histone H3 increased after addition of recombinant Aurora binding domains of different TPX2 like proteins. Radioactive kinase assay was performed with the first 100 amino acids at the N-terminus of TPX proteins.
