# Supplementary material for: Functional Divergence of Microtubule-Associated TPX2 Family Members in Arabidopsis thaliana
Source: Int J Mol Sci. 2020 Mar 22;21(6):2183. doi: 10.3390/ijms21062183 (PMC7139753; doi:10.3390/ijms21062183)
Supplement: Supplementary file 1 [file ijms-21-02183-s001.zip › Figure S4.pdf]

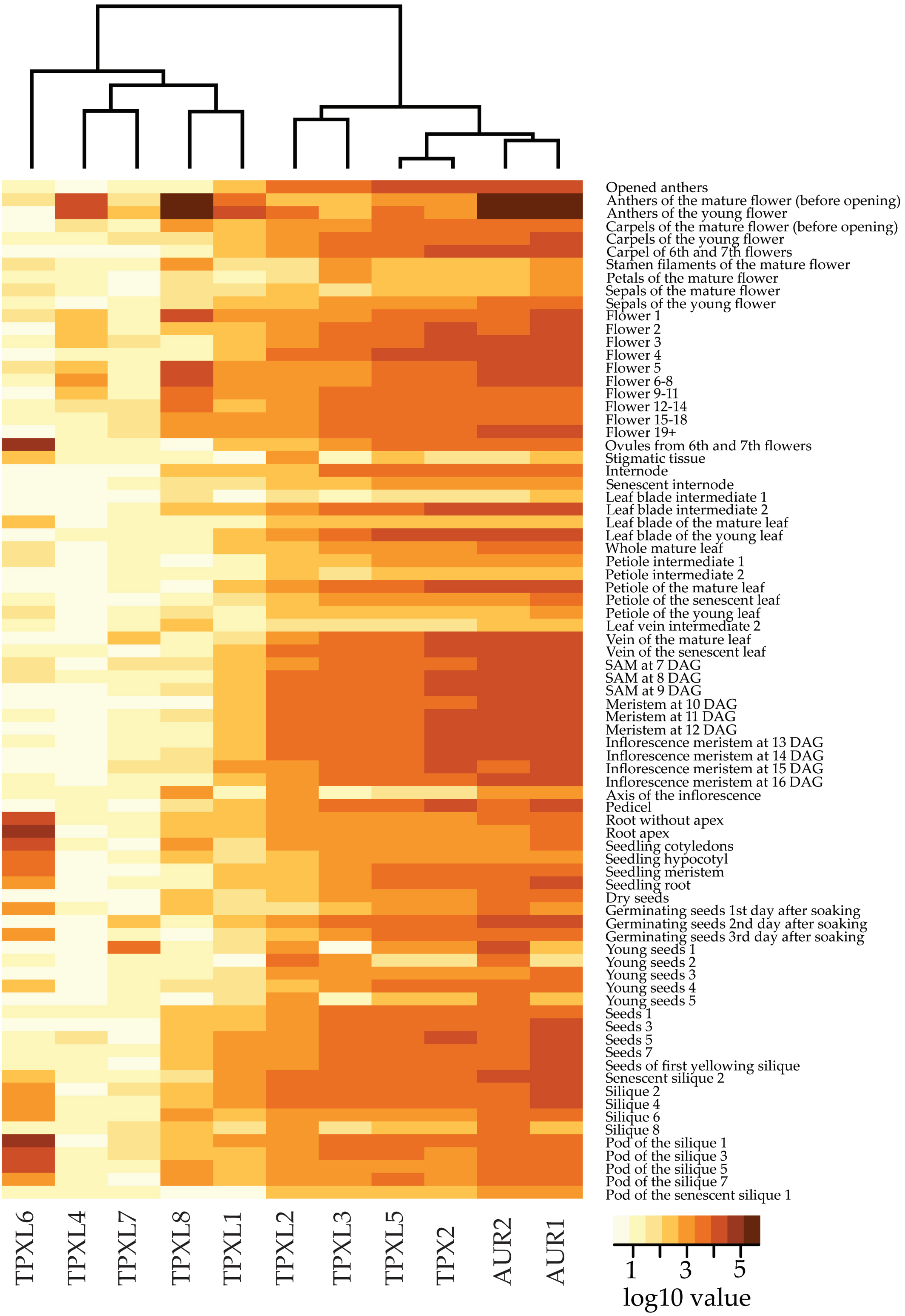

Figure S4: Expression analyses of TPXL and α-Aurora gene family during *Arabidopsis* development. Heat map and hierarchical cluster display differential expression profiles across various developmental stages (extended version of Figure 3). The color bar represents log10 expression values inferred from raw counts of [25]; thereby white colour representing the lowest expression values and brown signifies the highest expression level. The dendrogram was computed and reordered based on gene expression values.
