# Supplementary material for: Functional Divergence of Microtubule-Associated TPX2 Family Members in Arabidopsis thaliana
Source: Int J Mol Sci. 2020 Mar 22;21(6):2183. doi: 10.3390/ijms21062183 (PMC7139753; doi:10.3390/ijms21062183)
Supplement: Supplementary file 1 [file ijms-21-02183-s001.zip › Figure S5.pdf]

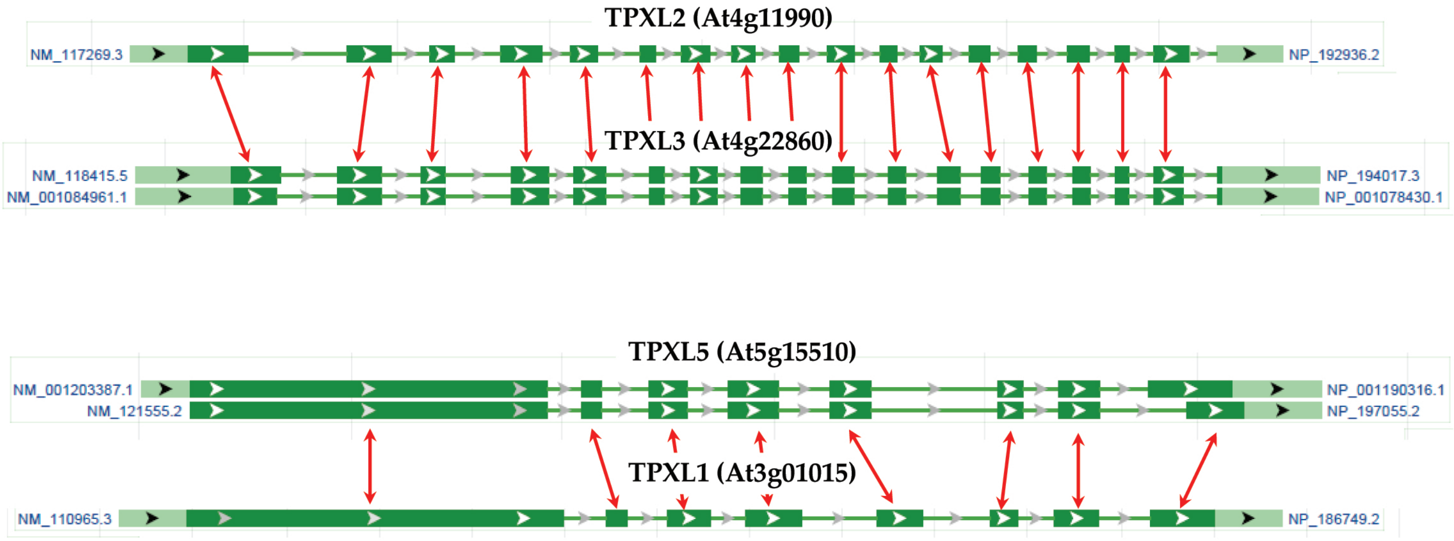


**Figure S5:** Similar exon-intron structure of some of the TPXL genes. Examples of gene duplications based on high similarity in the exon-intron structure of investigated genes, according to NCBI Database.
